# Study of obesity research using machine learning methods: A bibliometric and visualization analysis from 2004 to 2023

**DOI:** 10.1097/MD.0000000000039610

**Published:** 2024-09-06

**Authors:** Xiao-wei Gong, Si-yu Bai, En-ze Lei, Lian-mei Lin, Yao Chen, Jian-zhong Liu

**Affiliations:** a Wuhan Hospital of Traditional Chinese Medicine, Wuhan, China; b Hubei University of Chinese Medicine, Wuhan, China; c Hubei Provincial Hospital of Traditional Chinese Medicine, Wuhan, China; d Affiliated Hospital of Hubei University of Chinese Medicine, Wuhan, China; e Hubei Academy of Traditional Chinese Medicine, Wuhan, China.

**Keywords:** bibliometric, CiteSpace, machine learning, obesity, visual analysis, VOSviewer

## Abstract

**Background:**

Obesity, a multifactorial and complex health condition, has emerged as a significant global public health concern. Integrating machine learning techniques into obesity research offers great promise as an interdisciplinary field, particularly in the screening, diagnosis, and analysis of obesity. Nevertheless, the publications on using machine learning methods in obesity research have not been systematically evaluated. Hence, this study aimed to quantitatively examine, visualize, and analyze the publications concerning the use of machine learning methods in obesity research by means of bibliometrics.

**Methods:**

The Web of Science core collection was the primary database source for this study, which collected publications on obesity research using machine learning methods over the last 20 years from January 1, 2004, to December 31, 2023. Only articles and reviews that fit the criteria were selected for bibliometric analysis, and in terms of language, only English was accepted. VOSviewer, CiteSpace, and Excel were the primary software utilized.

**Results:**

Between 2004 and 2023, the number of publications on obesity research using machine learning methods increased exponentially. Eventually, 3286 publications that met the eligibility criteria were searched. According to the collaborative network analysis, the United States has the greatest volume of publications, indicating a significant influence on this research. coauthor’s analysis showed the authoritative one in this field is Leo Breiman. *Scientific Reports* is the most widely published journal. The most referenced publication is “R: a language and environment for statistical computing.” An analysis of keywords shows that deep learning, support vector machines, predictive models, gut microbiota, energy expenditure, and genome are hot topics in this field. Future research directions may include the relationship between obesity and its consequences, such as diabetic retinopathy, as well as the interaction between obesity and epidemiology, such as COVID-19.

**Conclusion:**

Utilizing bibliometrics as a research tool and methodology, this study, for the first time, reveals the intrinsic relationship and developmental pattern among obesity research using machine learning methods, which provides academic references for clinicians and researchers in understanding the hotspots and cutting-edge issues as well as the developmental trend in this field to detect patients’ obesity problems early and develop personalized treatment plans.

## 1. Introduction

Machine learning (ML), an essential subdivision of artificial intelligence (AI), encompasses a wide range of techniques and methodologies dedicated to developing models and algorithms that make computers acquire information from data to identify patterns, extract insights, and make informed decisions, all with the ultimate goal of improving future performance. The capacity of a machine to acquire knowledge from a given set of training data and subsequently make predictions for external data points from the original training data set is ML. With the development of interdisciplinary disciplines, ML has applications in multiple fields, ranging from medicine and more. Various learning methods, such as deep learning or neural networks, decision trees, as well as computer vision and natural language processing, are extensively utilized in disease research. These methods will soon become an essential part of a physician’s daily practice, acting as an enabler rather than a competitor.^[1]^

Obesity, a multifactorial and complex health condition, has emerged as a significant global public health concern. The main pathological manifestations of obesity are excessive fat deposition and metabolic disorders, the pathogenesis of which is related to genetic, environmental, dietary, exercise, psychological, and disease factors. Obesity not only poses immediate health risks but is also intricately linked to a myriad of chronic conditions, including diabetes, cardiovascular diseases, and multiple types of cancers.^[2]^ The study predicts that by 2025, 18% of men and 21% of women worldwide will be diagnosed with obesity.^[3]^ Conventional approaches to obesity management often face limitations in accurately predicting individual risk, tailoring interventions, and optimizing treatment outcomes. The inadequacy of a one-size-fits-all paradigm in obesity management underscores the need for innovative approaches that harness the possibility of ML algorithms as a transformative instrument for exploring predictive modeling, personalized interventions, clinical decision support systems, medical imaging, patient communication, and behavioral modification. With its rising prevalence and associated comorbidities, effective strategies for prevention, early identification, and therapeutic options are paramount.^[4]^

Applying ML techniques to obesity research holds tremendous promise, especially in the screening, diagnosis, and analysis of obesity.^[5]^ Despite the promising potential, research in this field still confronts several issues and challenges, such as data quality and quantity, privacy and security, interpretability of models, generalization across populations, clinical applicability, long-term effects and interventions, causality, and intervention analysis.^[6]^ As an emerging interdisciplinary field, the centers of much research have been employing ML algorithmic approaches to study obesity in relation to nutritional, environmental, social factors, genomic or genetic, and the intestinal microbiota in the last 20 years.^[7]^ But it is challenging to capture current trends and hotspots. The studies such as reviews and meta-analyses cannot provide trend predictions.

Bibliometrics integrates mathematical, statistical, and bibliographical knowledge systems and focuses on the quantitative study of information, which includes publications, journals, authors, references and keywords, countries/regions, and institutions’ cooperation analysis. Bibliometrics aims to illustrate the current situation of all scientific study dimensions as well as demonstrate communication and collaboration in academia.^[8,9]^ It provides a reliable basis for understanding publication patterns, partnerships, and impact within a particular field or discipline. The application of bibliometrics in medicine helps people analyze a large number of publications both holistically and in terms of specific aspects to capture trends and hotspots.^[10]^ For interdisciplinary research, this research method is very suitable and is currently widely used in the study of ML in disease.^[11]^ However, there have been an absence of bibliometric analysis using ML methods in obesity study. This analysis intends to conduct the bibliometric approach to visualize and analyze scholarly articles about obesity research using ML methods published between 2004 and 2023 for the first time. To assist researchers in knowledge discovery and overview, to uncover the research hotspots, evolutionary trends and intrinsic links between related studies in this field, and to promote inter-disciplinary cooperation and exchanges.

## 2. Materials and methods

### 2.1. Data sources and search strategies

**Figure 1.**
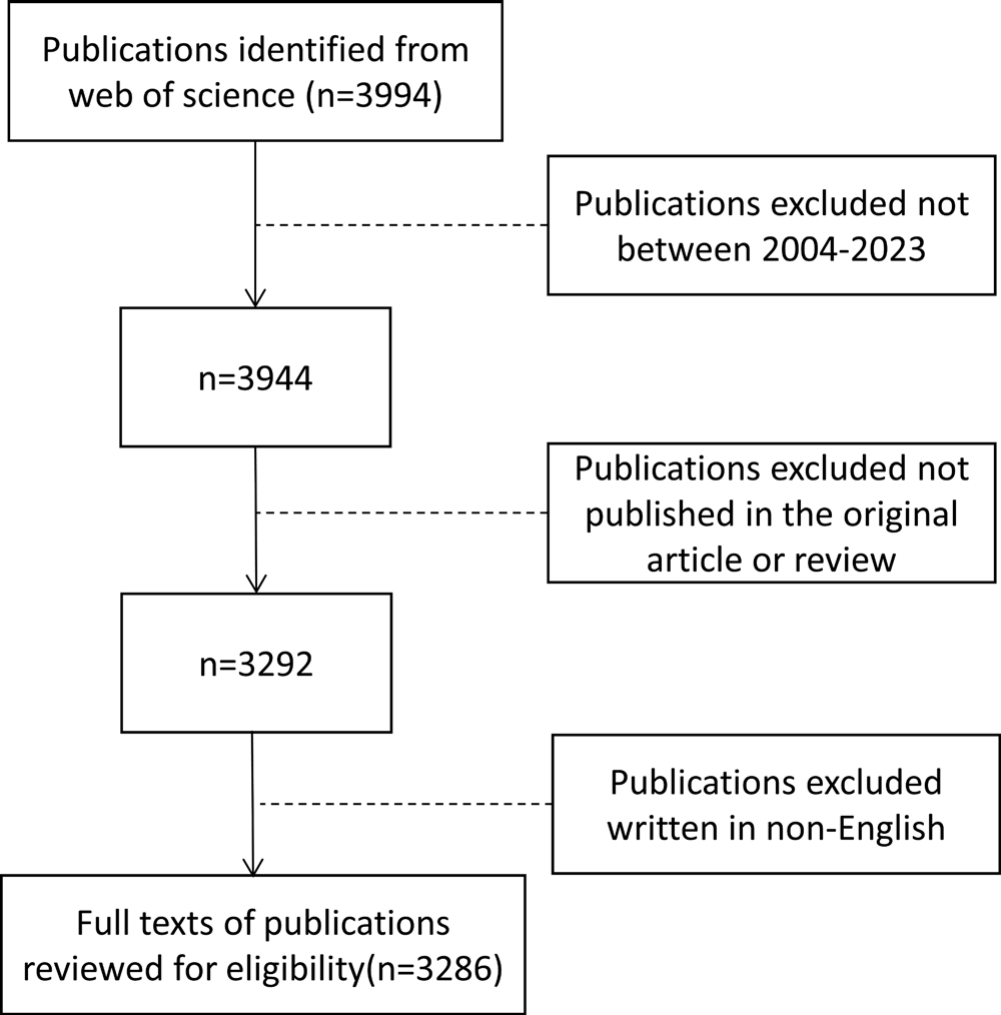
Screening flow chart.

The Web of Science core collection (WOSCC) was the primary database source for the publications on obesity research using ML methods, which is the most reliable, numerous, and high-impact citation database for conducting bibliometric studies.^[12]^ The Topic Search (TS) approach is used in WOSCC to retrieve publications, combined with the subject words in the MeSH word list. The search formula is as follows: TS=(“Machine learning” OR “Naive Bayes” OR “Decision trees” OR “Random forest” OR “Support vector machines” OR “Gradient boosting decision tree” OR “Adaptive boosting” OR “Extreme gradient boosting” OR “Light gradient boosting machine” OR “Categorical boosting” OR “Generalized additive model” OR “Artificial neural networks” OR “Data Mining” OR“Deep learning”) AND TS=(“obesity”OR“overweight”OR “obes*” OR “over$weight”OR “high-fat diet”OR “body mass index” OR “BMI”). The search was terminated on January 9,2023, to prevent data bias caused by database changes, and furthermore, limited the period from January 1, 2004, to December 31, 2023. AS Shown in Figure 1, the document types were limited to both “article” and “review,” excluded other types, and only English was accepted in terms of language. Finally, 3286 articles were retrieved and exported by using plain text files to derive relevant information such as authors, institutions, keywords, citations, et cetera (etc.).

### 2.2. Data analysis and visualization

Excel, CiteSpace, and VOSviewer are the primary software utilized. The most commonly available software for analyzing bibliometrics is CiteSpace, which Chaomei Chen created.^[13]^ We utilized CiteSpace (6.2.R4) Advanced edition to visualize and analyze the publications. The output mainly includes reference collaboration, keyword timeline graphs, reference timeline graphs, dual-map overlay of journals, and citation bursts. VOSviewer is a reliable and flexible software for bibliometric network analysis and visualization.^[14]^ Countries and institutions’ cooperation analysis, coauthors analysis, co-occurring keywords analysis, and co-cited reference analysis were visually analyzed in VOSviewer (1.6.20). Additionally, we used Microsoft Excel 2021 to analyze and graph the volume of publications and citation frequency in this field. Since each raw data utilized in this study was retrieved from publicly accessible databases, no ethical review was required. The pertinent material was visualized as knowledge maps, and the crucial nodes and connections between the various maps were deciphered and examined.

## 3. Results

### 3.1. Analysis of annual publications and citations

**Figure 2.**
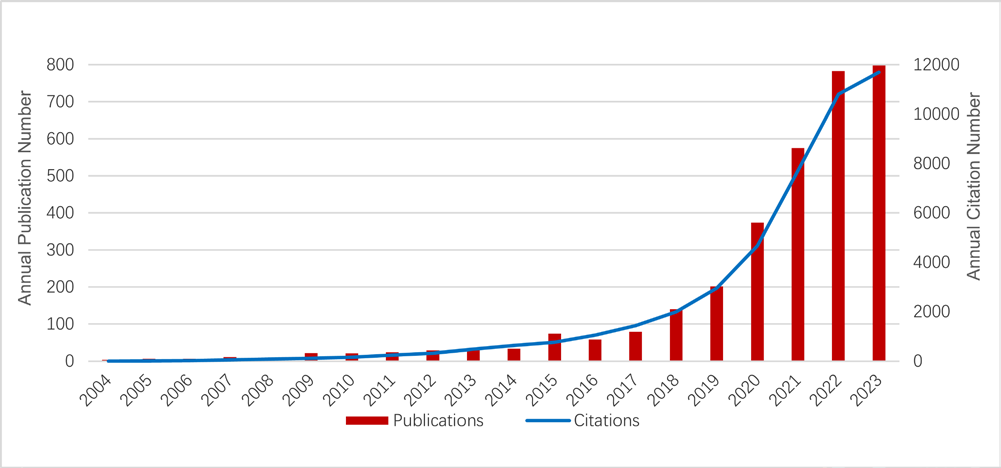
Analysis of annual publications and citations.

Based on the selection criteria, 3286 English articles published between 2004 and 2023 were included, as illustrated in Figure 1. Figure 2 illustrates the total volume of publications and the rate of citations each year. An overall pattern for publications on obesity research using ML methods has shown remarkable growth year by year. However, between 2016 and 2017, the volume of publications declined slightly. There was a logarithmic increase in citation frequency of obesity and ML-related research, especially between 2015 and 2016, 2020 and 2021, and 2021 and 2022. The highest citation frequency was in 2023, totaling 11,699 citations.

### 3.2. Analysis of major countries/regions

**Figure 3.**
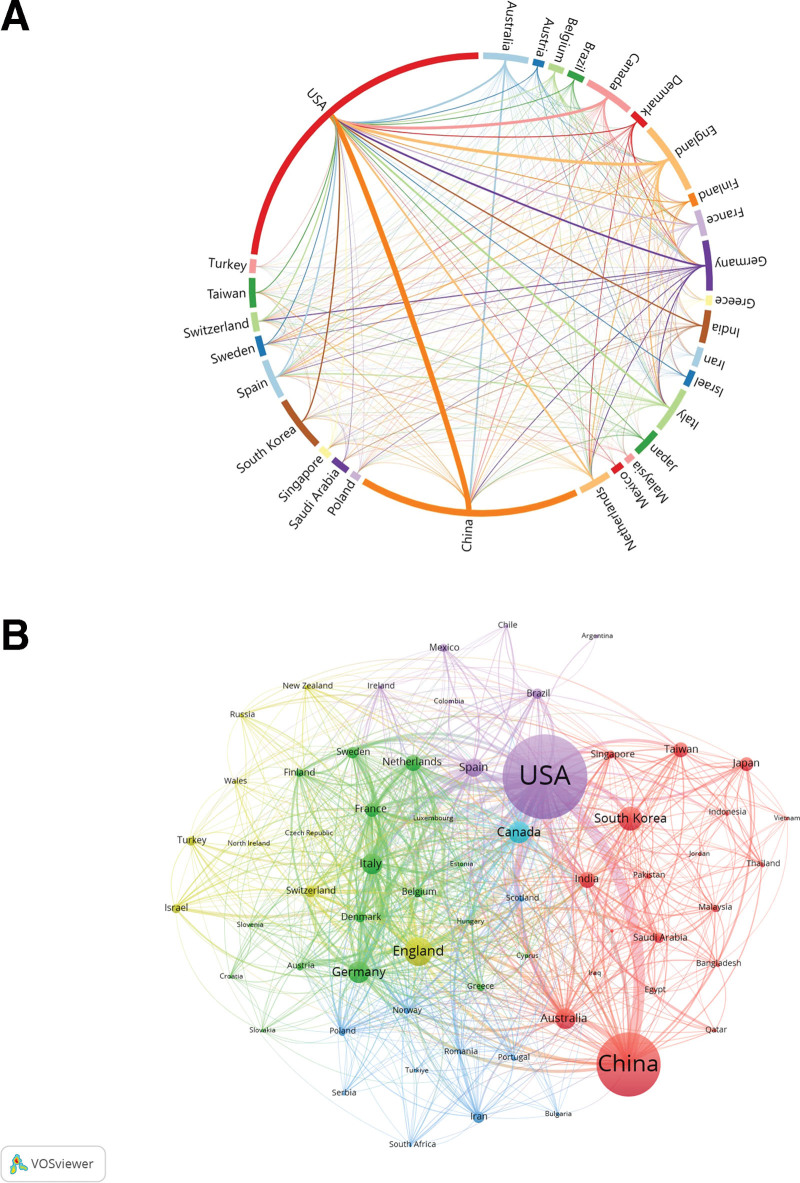
Analysis of country/region in the field of obesity research using machine learning methods. (A) Countries/regions involved in obesity research using machine learning methods, The links between countries/regions indicate their collaborations and connections. (B) Analysis of collaborative network visualization of countries/regions in VOSviewer. The figure shows the countries/regions with more than 5 number of documents. The nodes of different colors represent the countries/regions with different clusters, and the size of the nodes indicates their node sizes.

The analysis shows that 118 countries/regions studied obesity using ML methods. The specifics of the top 10 countries/regions are listed in Table 1, including the volume of publications, citation frequency, and centrality. In summary, in terms of both volume of publications and frequency of citations, the United States of America (USA) ranked first (1114), followed by China (755) and England (247). Regarding citation frequency, the USA occupies the first place (19,211). With 6898 publications, China came in second. The top 30 countries/regions and international partnerships, sorted by publication volume, are presented in Figure 3A. As indicated in the figure, the associations between countries/regions are centered among the United States and China, the United States and England, the United States and Canada, the United States and Australia, and the United States and Germany, with the strongest associations between the United States and China.

**Table 1:** The top 10 countries/regions including the volume of publications, citation frequency, and centrality.

Rank	Countries/regions	Publications	Citations	Centrality
1	USA	1114	19,211	0.15
2	China	755	6898	0.07
3	England	247	5938	0.13
4	Korea	188	2053	0.04
5	Germany	172	2883	0.06
6	Canada	170	2757	0.16
7	Italy	157	2778	0.11
8	Australia	155	2130	0.17
9	Spain	136	1591	0.09
10	India	113	885	0.08

USA = United States of America.

According to the proximity of cooperation, the country and region are broadly classified into 6 clusters based on the analysis conducted by VOSviewer software. Different colors represent different clusters, with larger nodes representing a larger volume of documents. As shown in Figure 3B, the most notable obesity research using ML methods was conducted in the United States and China, and they have close collaboration with many other countries, including Korea, England, Canada, Germany, Australia, Spain, as well as Italy.

### 3.3. Analysis of institutions

The specifics of the top 10 institutions are presented in Table 2, including the volume of publications, citation frequency, and centrality. In terms of publication amount, Harvard Medical School (83) stands first, then Stanford University (55) and the University of California, San Francisco (43). The USA has 5 institutions while China has 4 among the top 10 in regard to publication volume. The Weizmann Institute of Science (1975) is the most frequently referenced institution; Tel Aviv University (1927) and Tel Aviv Sourasky Medical Center (1829) come in the second and third. In addition, The USA has 7 of the top 10 most referenced institutions, followed by Israel with 3.

**Table 2:** The top 10 institutions including the volume of publications, citation frequency, and centrality.

Rank	Institution	Publications	Citations	Centrality
1	Harvard Medical School	83	1114	0.03
2	Stanford University	55	1036	0.02
3	University of California, San Francisco	43	798	0.04
4	Capital Medical University	39	309	0.01
5	University of California, Los Angeles	39	873	0.01
6	Peking University	38	380	0.03
7	Seoul National University	38	162	0.00
8	Shanghai Jiao Tong University	37	368	0.00
9	Mayo Clinic	35	719	0.02
10	Sun Yat-sen University	34	323	0.01

Typically, centrality ≥ 0.1 indicates that the node is central in a field. Remarkably, the University of Texas System (0.12, 2005), the University of Pennsylvania (0.11, 2019), the University of Washington Seattle (0.10, 2015), the Stanford University (0.10, 2015), Chinese Academy of Sciences (0.10, 2017), and plenty of additional institutions demonstrate considerable centrality, indicating that these institutions hold a prominent place in obesity research using ML methods.

**Figure 4.**
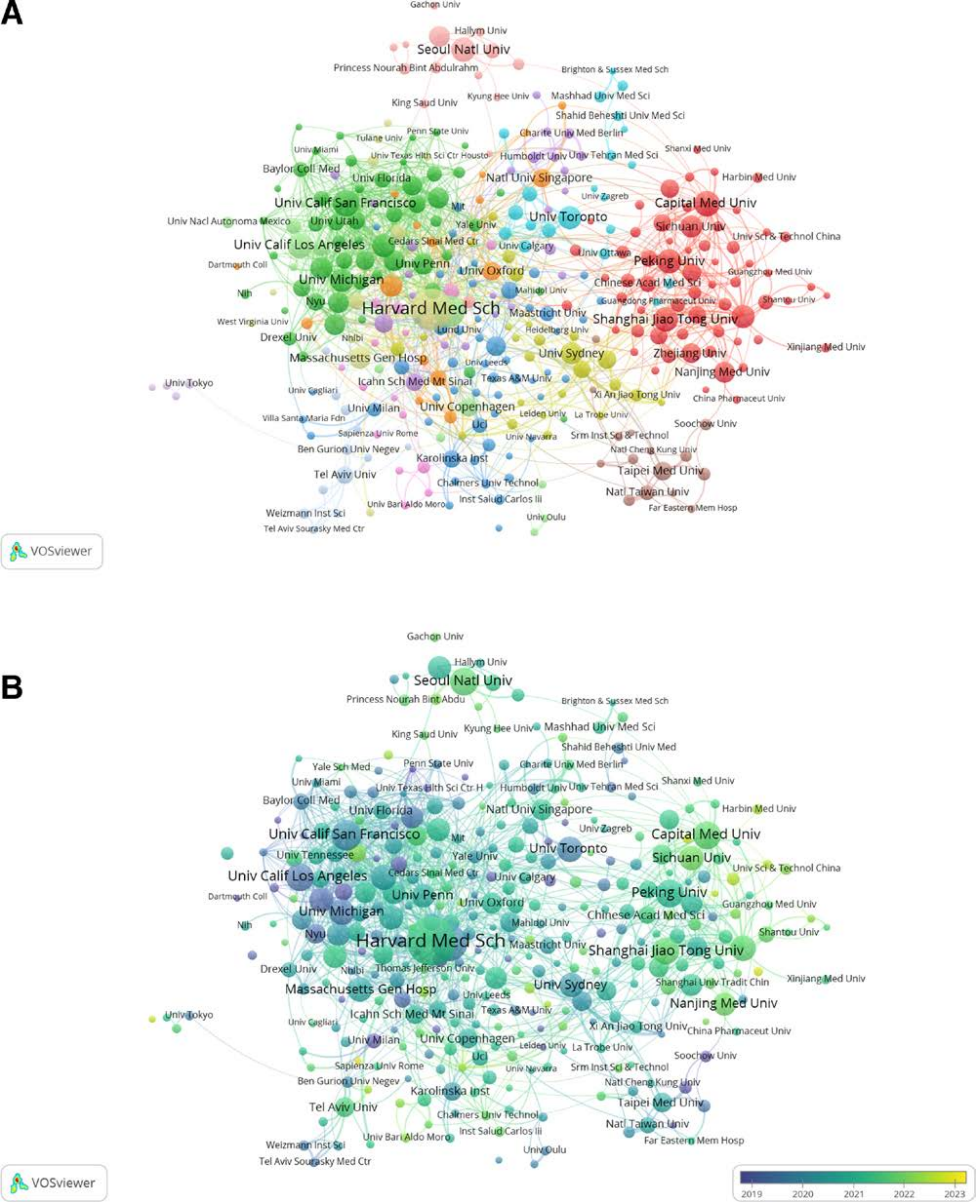
Analysis of institution in the field of obesity research using machine learning methods. (A) Visual analysis of organization collaboration network in VOSviewer. The figure shows the institutions with more than 6 documents. The nodes of different colors represent the institutions of different clusters, and the size of the nodes indicates the frequency of their occurrence. (B) Visual analysis of the number of articles published by institutions in the past 5 years. The color bias towards yellow means a higher ratio of publications, the color bias towards purple means a lower ratio.

In VOSviewer, an analysis of organization collaboration network visualization (Fig. 4A) and overlay visualization of the quantity of papers published by establishments in the past 5 years (Fig. 4B) is conducted. These can provide a reference point for researchers seeking institutional collaboration in obesity research using ML methods. As Figure 4B shows, the different colors reflect the evolution of research trends in obesity research using ML methods, with those closer to yellow indicating that these institutions are new to the field and have a higher volume of recent publications. Conversely, those closer to purple indicate a smaller ratio in the past 5 years. Figure 3B reveals that Harvard Medical School, Shanghai Jiao Tong University, Capital Med University, and Seoul National University are at the top of the list with regard to the percentage of papers published throughout the last 5 years, while institutions, for example, the University of California, San Francisco, University of California, Los Angeles, and the University of Michigan have recently completed lesser research.

### 3.4. Analysis of active authors

Two or more authors are stated to establish a co-citation connection if they are cited in one or more subsequent publications at the same time. Suppose these 2 authors have a higher co-citation frequency. In that case, it means that they have a closer academic relationship.^[15]^ Co-cited authorship analysis of obesity research using ML methods can not only be used to reveal the development status and scientific structure but also be used to carry out frontier and scientific research evaluation, to provide advanced support for macro scientific and technological decision making. The specifics of the top 10 authors are illustrated in Table 3, including the volume of publications, co-citation rate, institutions, and the total link strength. Kim, Jong Yeol (Korea Inst Oriental Med, Korea) (11) and Lee, Bum Ju (Korea Inst Oriental Med, Korea) (11) are the authors who have the top publication volume, followed by Schwab, Joseph H. (Harvard Medical School, USA) (10), Kunze, Kyle N. (Hospital for Special Surgery, USA) (10), and Grossi, Enzo (Centro Diagnostico Italiano, Italy) (10).

**Table 3:** Top 10 authors in terms of publications, co-citation rate, institutions, and the total link strength.

Rank	Author	Publications	Institutions	Total link strength
1	Kim, Jong Yeol	11	Korea Inst Oriental Med (Korea)	17
2	Lee, Bum Ju	11	Korea Inst Oriental Med (Korea)	17
3	Schwab, Joseph H.	10	Harvard Med Sch (USA)	55
4	Kunze, Kyle N.	10	Hosp Special Surg (USA)	28
5	Grossi, Enzo	10	Centro Diagnostico Italiano (Italy)	5
6	Segal, Eran	9	Weizmann Inst Sci (Israel)	37
7	Nwachukwu, Benedict U.	9	Hosp Special Surg (USA)	34
8	Polce, Evan M.	9	Univ Wisconsin (USA)	25
9	Kwon, Young-Min	9	Harvard Med Sch (USA)	22
10	Ma, Jun	9	Peking Univ (China)	20

USA = United States of America.

**Figure 5.**
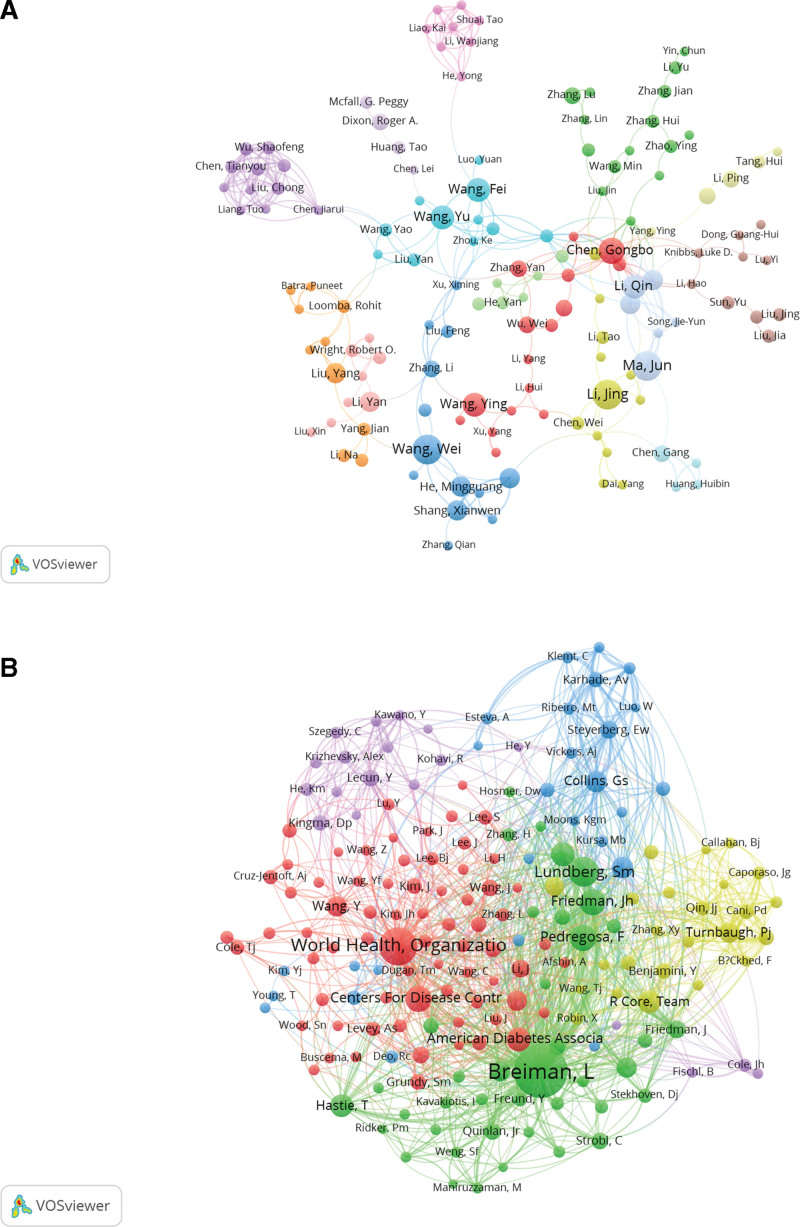
Analysis of author in the field of obesity research using machine learning methods. (A) Visual analysis of author collaboration network in VOSviewer. The figure shows the authors with more than 3 documents. The nodes in different colors represent the authors in different clusters, and the size of the nodes indicates the frequency of their occurrence. (B) Visual analysis of collaboration network of authors’ citations in VOSviewer. The size of the nodes indicates the frequency oil their occurrence.

In VOSviewer, an analysis of the authors’ partnerships of obesity research using ML methods associated with publications is displayed in Figure 5A. Chen Gongbo and Wang Yu have larger nodes, indicating that they have made more significant contributions to the authors’ partnership. Chen Gongbo is connected to Wang Fei, Wang Min, Li Ping, Knibbs Luke D, Li Qin, and Fuchs Tobia A, while Wang Yu cooperates closely with Chen Jiarui, Dixon Roger A, He Yong, Xu Ximing, and Loomba Rohit. A network diagram of co-cited authors (Fig. 5B) reveals that Leo Breiman (University of California, Berkeley, USA) (542) is the top-ranked co-cited author, the World Health Organization (275) comes second. Varying colors distinguish and 5 primary clusters of authors, including Leo Breiman, Lundberg Sm, etc. (green); World Health Organization, Centers for Disease Control and Prevention, etc. (red); Knight Js, Collins Gs, Steyerberg Ew, etc. (blue); Turnbaugh Pj, Core Team, etc. (yellow); Lecun Y, Kingma Dp, etc. (purple).

### 3.5. Analysis of journal distribution and research areas

By analyzing journals using bibliometrics, we can identify the critical journals in obesity research using ML methods related to these fields. Impact factor and Journal Citation Report partitions are included in Table 4, which presents the top 10 journals ranked by the volume of publications in the field. The Impact factor and Journal Citation Report quartiles indicate the impact of the journal.

**Table 4:** Impact factor and Journal Citation Report partitions of the top 10 journals.

Rank	Journal	Publications	IF (JCR 2022)	JCR quartile
1	Scientific Reports	119	4.6	Q2
2	PLOS One	91	3.7	Q2
3	Nutrients	38	5.9	Q1
4	International Journal of Environmental Research and Public Health	37	4.614(2021)	Q2
5	Applied Sciences-based	37	2.7	Q3
6	Frontiers in Endocrinology	37	5.2	Q1
7	IEEE Access	33	3.9	Q2
8	Sensors	31	3.9	Q2
9	Diagnostics	30	3.6	Q2
10	Frontiers in Public Health	29	5.2	Q1

IF = Impact Factor, JCR = Journal Citation Report.

**Figure 6.**
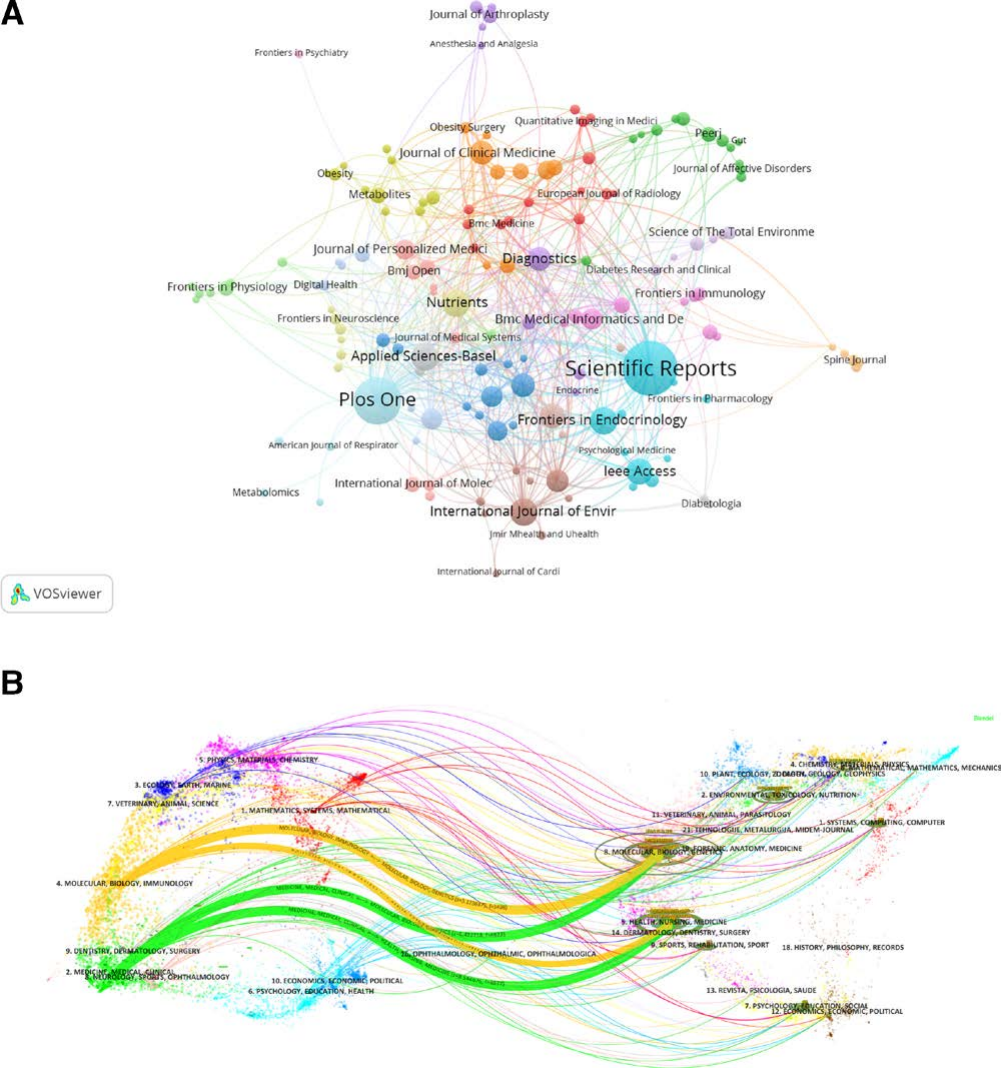
Analysis of journal in the field of obesity research using machine learning methods. (A) Visual analysis of journals collaboration network in VOSviewer. The figure shows the journals with more than 4 documents. The nodes indifferent colors represent the journals in different clusters, and the size of the nodes indicates the frequency of their occurrence. (B) The dual-map overlay of journals. The labels on the left represent citing journals, the labels on the right represent cited journal, and colored paths indicate citation relationships.

In VOSviewer, an analysis of the journals’ partnerships in obesity research using ML methods associated with publications is displayed in Figure 6A. According to the proximity of cooperation, the journals are categorized into about 9 clusters. As shown in the Figure 6A, research in the light blue cluster centers on multidisciplinary approach; research in the green cluster centers on neurology; research in the yellow cluster centers on nutrient; research in red cluster centers on iconography; research in purple cluster centers on sports medicine; research in orange cluster centers on clinical medicine; research in pink cluster centers on computer and information technology; the blue cluster, as well as the brown cluster are connected to multiple clusters, demonstrating that they are issues which extend across all research directions.

To analyze the development of academic citation and co-citation between citing and cited journals, we utilized the dual-map overlay of scholarly journals in CiteSpace (Fig. 6B).^[16]^ On the left of the figure is a collection of citing journals, representing the current knowledge front, and on the right is a collection of cited journals, representing the knowledge foundation of the research area. The connecting lines between them reflect the macro structural development pattern of the field, including patterns of independence, divergence, convergence, and cross-cutting.^[17]^ As seen in the figure, the obesity research using ML methods represents a cross-cutting development pattern.

### 3.6. Analysis of keyword co-occurrence and burst

Keyword co-occurrence analysis can characterize research hot topics and burst keywords can symbolize research frontiers of obesity research using ML methods. The keyword, occurrences, and total link strength divisions are included in Table 5, which lists the top 20 keywords based on the occurrences in the field. The top-ranking keyword is “ML” (1037), followed by “obesity” (307), and deep learning (211). Furthermore, “AI” (170), “predictive models” (128), and “body mass index” (111) are also main keywords, as hot topics in obesity research using ML methods.

**Table 5:** Top 20 keywords in terms of occurrences, and total link strength.

Rank	Keyword	Occurrences	Total link strength
1	machine learning	1037	2123
2	obesity	307	788
3	deep learning	211	426
4	artificial intelligence	170	424
5	predictive models	128	308
6	body mass index	111	268
7	data mining	107	242
8	prediction	104	268
9	random forest	99	219
10	risk factor	96	237
11	classification	86	242
12	biomarker	75	174
13	type 2 diabetes	72	146
14	diabetes	69	215
15	metabolic syndrome	68	155
16	gut microbiota	62	123
17	artificial neural network	59	128
18	hypertension	56	142
19	support vector machines	52	127
20	covid-19	51	129

**Figure 7.**
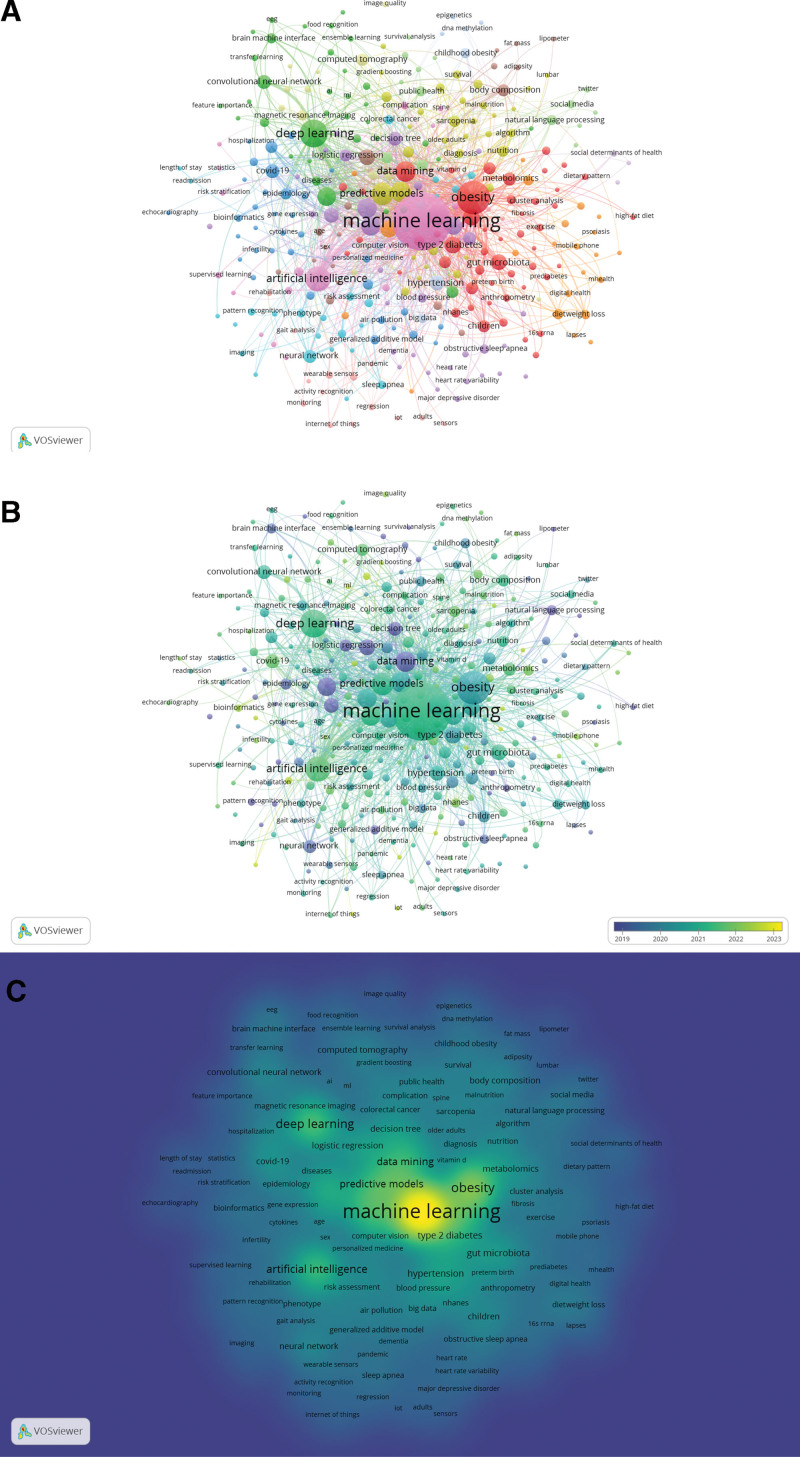
Analysis of keyword in the field of obesity research using machine learning methods in VOsviews. (A) Visual analysis of keywords collaboration network. The figure shows the keywords with more than 5 occurrences, different colors represent different clusters, and the size of nodes indicates their frequency, (B) Visual analysis of the frequency of keywords in recent 5 years. The color bias towards yellow means a higher ratio of publications, the color bias towards purple means a lower ratio. (C) Visual analysis of the item density of institutional publications. The color bias towards yellow means a higher ratio, the color bias towards blue means a lower ratio.

In VOSviewer, an analysis of the keyword co-occurrence of obesity research using ML methods is presented in Figure 7A. About 5 clusters emerge by the keywords based on the proximity of cooperation. The purple cluster corresponds to computer science and technology (including ML, AI, decision trees). The red cluster corresponds to the object of obesity research (such as obesity, type 2 diabetes, gut microbiota, metabolomics, children) and obesity-related research methods of informatics (data mining, cluster analysis, etc.). The blue cluster corresponds to epidemiology (COVID-19, epidemiology, etc.). The green cluster corresponds to some specific methods of ML (deep learning, predictive models, convolutional neural networks, etc.). The yellow cluster corresponds to the prediction model and application in the clinic (including predictive models, diagnosis, computed tomography). The light blue cluster (such as neural network, phenotype) as well as the orange cluster (including diet, weight loss) are connected to multiple groups, proving that these problems affect various study areas. Figure 7B displays the frequency of the keyword in the last 5 years. The figure shows that “ML” and “AI” are recent research hotspots. On the contrary, “data mining” and “logistic regression” are relatively outdated.

Figure 7C shows the item density of publications in obesity research using ML methods in each keyword from 2004 to 2023. In item density visualization, every point has a color that represents the item’s density. In the chromatogram, the color change reflects the hotness of the keywords in the obesity research using ML methods. In the map of item density consisting of keywords, the areas with high volume and hotness are in yellow, and the areas with low volume and hotness are in blue. The results show that “ML,” “obesity,” “deep learning,” “body mass index,” and “predictive models” are relatively hot topics.

**Figure 8.**
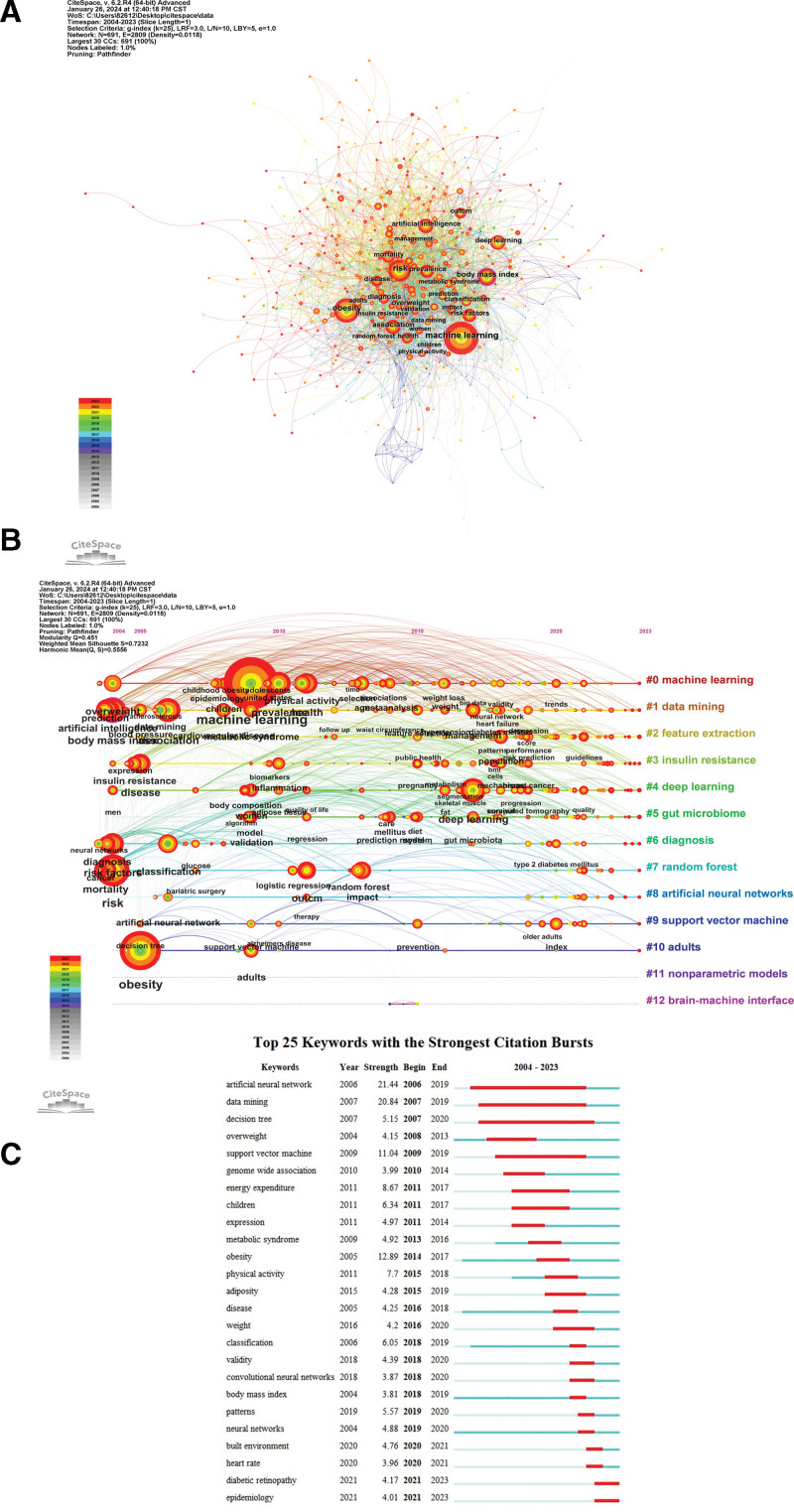
Analysis of keyword in the field of obesity research using machine learning methods in CiteSpace. (A) Visual analysis of keywords collaboration network. The color and thickness of the node inner circle represent the frequency of occurrence in different periods. (B) The timeline graph of keywords in CiteSpace. Each horizontal line represents a cluster. Nodes size reflects co-citation frequency, and the links between nodes indicate co-citation relationships. Nodes occurrence year is the time when they were first co-cited. (C) The top 25 keywords with the strongest citation bursts. The blue line indicates the time interval, and the red line indicates the period when the keyword burst occurs.

In CiteSpace, keywords can be visualized and analyzed, including contribution analysis, clustering analysis, and timeline graph analysis. The size of the node indicates the frequency of the keyword, the color and coarseness of the circle inside the node stands for the occurrence rate of the keyword in various stages of time, and if there is a purple ring outside the node, it means that the keyword has a high centrality (≥0.1).^[18]^ Figure 8A shows the keywords of diseases related to obesity research using ML methods, mainly diabetes, metabolic syndrome, insulin resistance, cardiovascular disease, cancer, etc. The code keywords of ML methods are ML, deep learning, AI, data mining, random forest, and prediction models. Among them, obesity, risk factors, association, body mass index, and artificial neural networks have better centrality.

The timeline graph in CiteSpace demonstrates the chronological ordering of keywords in the clusters by year of initial occurrence. As shown in Figure 8B, #0 (ML) is the most critical cluster. The keywords in this categorizing have changed over time, shifting from earlier keywords like overweight and ML to more cutting-edge ones like experience. Another critical foundation cluster is #1 (data mining). In this categorizing, keywords including obese patients, cohort study, heart diseases, and phenotypes are the current focus. Furthermore, during the evolution process, keywords are separated into an additional 11 groups, containing #2(feature extraction), #3 (insulin resistance), #4 (deep learning), #5 (gut microbiome), #6 (diagnosis), #7 (random forest), #8 (artificial neural networks), #9 (support vector machine), #10 (adults), #11 (nonparametric models), #12 (brain-machine interface).

Figure 8C displays the top 25 keywords with the strongest citation bursts, which indicate keywords with a sharp increase in citations over a certain period of time. The red segment shows the length of the period throughout which the keyword outburst, while the blue segment represents the time interval. The terms used frequently as time passes are called “Burst words.” Research edge expansion and shift can be inferred from the arrangement of the most periodically referenced keywords. According to the figure, the prior research focused primarily on artificial neural networks, data mining, and decision tree about overweight, obesity, and body mass index; between 2010 and 2020, support vector machine and pathological mechanisms about obesity was the main topic, including genome-wide association, energy expenditure, physical activity, adiposity; after 2020, the relationship between obesity and its comorbidities and complications is a hot research topic, including built environment, heart rate, diabetic retinopathy, and epidemiology. Visual analysis of relevant keywords helps to grasp the preoccupations and hotspots of obesity research using ML methods.

### 3.7. Analysis of co-cited reference

**Figure 9.**
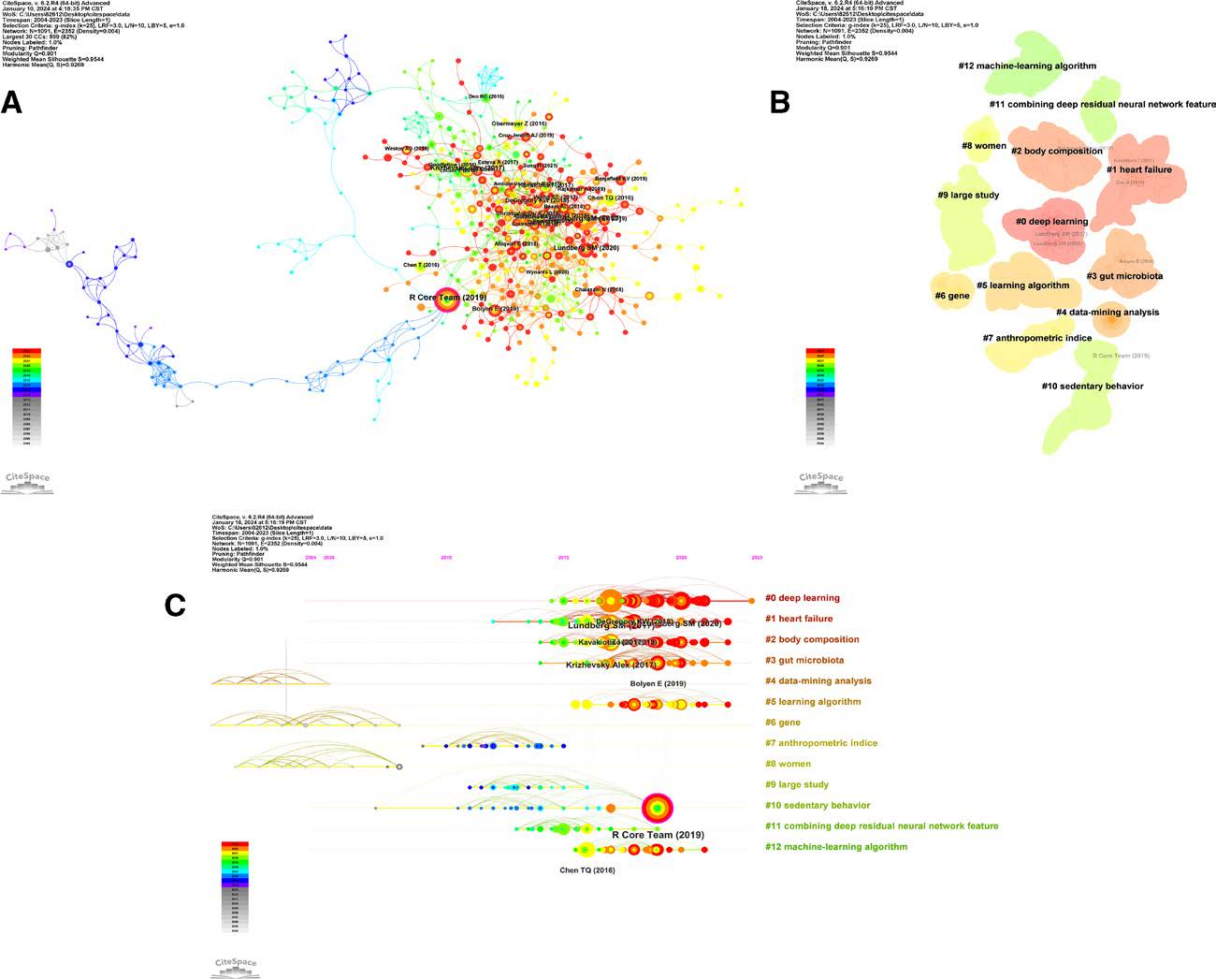
Analysis of reference in the field of obesity research using machine learning methods in CiteSpace. (A) Analysis of the network of references in Citespace. Node size is proportional to the number of times the article is co-cited. (B) Clustering of references based on similarity between references. The clustering is based on the degree of association between the literature and was divided into mainly 13 categories in different colors. (C) The timeline graph of keywords in CiteSpace. Each horizontal line represents a cluster. Nodes size reflects co-citation frequency, and the links between nodes indicate co-citation relationships. Nodes occurrence year is the time when they were first co-cited.

The highly cited reference analysis can help us quickly search for classic articles in obesity research employing ML methods, as displayed in Table 6 and Figure 9A, which illustrate the top 10 highly cited reference and their corresponding authors and source title. The article ranks first is “R: a language and environment for statistical computing” (R Core Team, et al, 2013) (100), which explores the origin of the R language, its main features, and importance in scientific research, data analysis, and applications. Furthermore, followed by “A Unified Approach to Interpreting Model Predictions” (Lundberg, S. M., et al, 2017) (64). Moreover, it also includes article “ImageNet classification with deep convolutional neural networks” (Krizhevsky, A., et al, 2017) (45), which offers an original method of identifying images via deep convolutional neural networks (CNNs). This article is a classic document on the utilization of deep learning techniques in images and provides a basis for subsequent medical research in the design of deep neural networks for picture identification and other computer vision applications.

**Table 6:** Top 10 highly cited references.

Rank	Article title	Total citation	Average citation per year	Source title	First author	Published year
1	R: a language and environment for statistical computing	100	10.00	R Foundation for Statistical Computing	R Core Team	2013
2	A unified approach to interpreting model predictions	64	10.67	ADV NEURAL INFORM PR	Lundberg, SM	2017
3	ImageNet classification with deep convolutional neural networks	45	7.50	Communications of the ACM	Krizhevsky, A	2017
4	From local explanations to global understanding with explainable AI for trees	42	14.00	NATURE MACHINE INTELLIGENCE	Lundberg, SM	2020
5	Predicting diabetes mellitus with machine learning techniques	31	6.20	FRONTIERS IN GENETICS	Zou, Q	2018
6	Machine learning and data mining methods in diabetes research	31	5.17	COMPUT STRUCT BIOTEC	Kavakiotis, I	2017
7	Reproducible, interactive, scalable and extensible microbiome data science using QIIME 2	31	7.75	NAT BIOTECHNOL	Bolyen, E	2019
8	XGBoost: a scalable tree boosting system	29	4.14	KDD’16: PROCEEDINGS OF THE 22ND ACM SIGKDD INTERNATIONAL CONFERENCE ON KNOWLEDGE DISCOVERY AND DATA MINING	Chen, TQ	2016
9	A review of machine learning in obesity	28	5.60	OBESITY REVIEWS	DeGregory, KW	2018
10	A systematic review shows no performance benefit of machine learning over logistic regression for clinical prediction models	24	6.00	JOURNAL OF CLINICAL EPIDEMIOLOGY	Christodoulou, E	2019

Article co-citation analysis reveals the influence and importance of a reference in the field by analyzing the citations, and uncovers vital information about hotspots, academic collaborations, and research paths in the research field.^[19]^ As displayed in Figure 9B, the co-citation analysis in CiteSpace presents the articles are divided into 13 main clusters displayed in various colors according to the degree of relevance and frequency of citations. #0 (deep learning) is an especially significant cluster. In the timeline graph of co-citation analysis (Figures 9C), 3 clusters represent the initial domains in obesity research utilizing ML methods, including #4 (data-mining analysis), #6 (gene), and#8 (women), while during about 2010 to 2015, #7 (anthropometric indice), #9 (large study), and #10 (sedentary behavior) start to increase. After 2015, a large number of publications emerge, #0 (deep learning), #1 (heart failure), #2 (body composition), #3 (gut microbiota), #5 (learning algorithm), and #12 (machine-learning algorithm) are the hot research areas.

**Figure 10.**
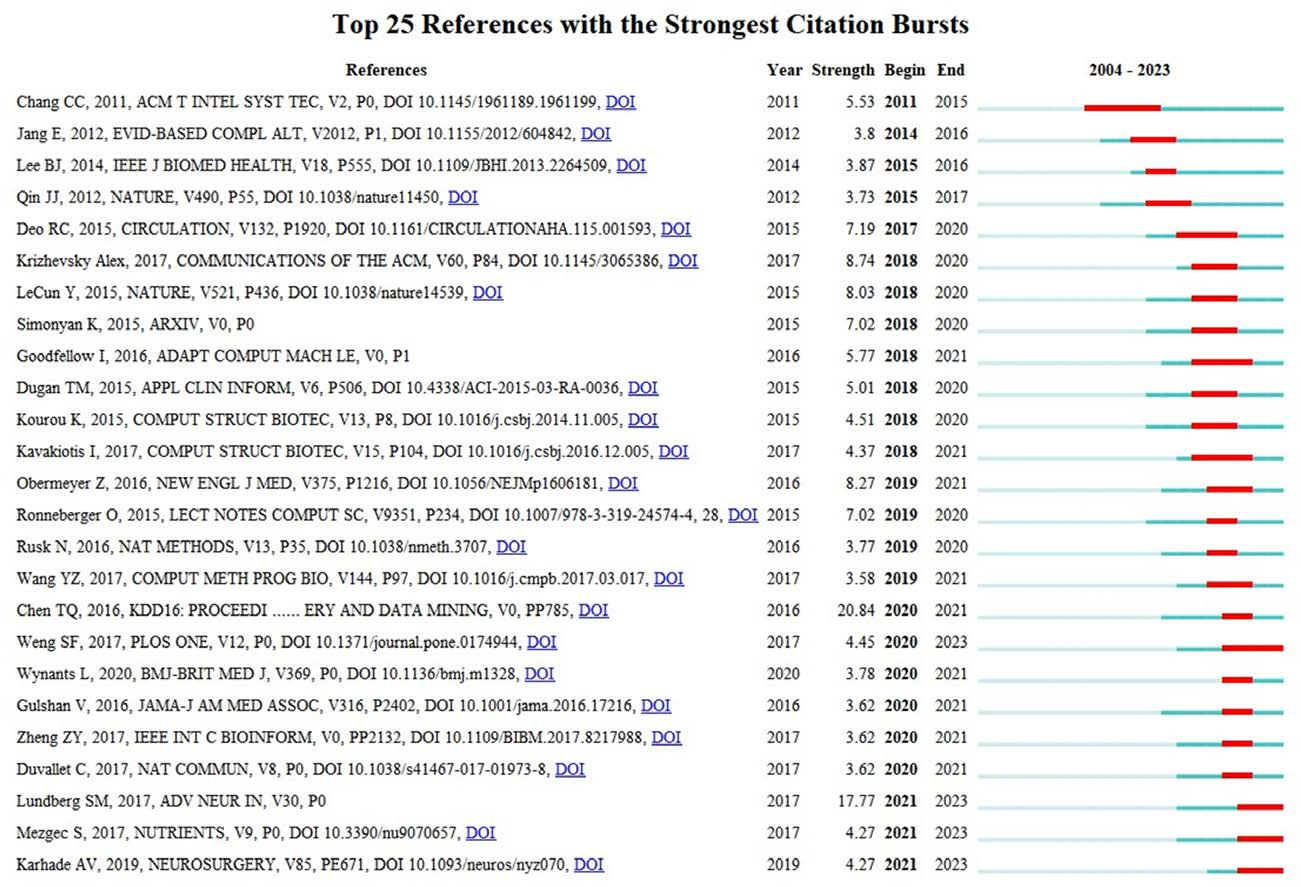
The top 25 references with the strongest citation bursts. The blue line indicates the time intervals, and the red line indicates, the period when the keyword burst occurs.

The top 25 references with the strongest citation bursts not only underscore their significance in advancing knowledge but also serve as guiding beacons for future research endeavors. The analysis with the CiteSpace were shown in Figure 10, among them, 9 references are for the topics of ML methods and technology, such as, deep learning, prediction models, Xgboost, convolutional networks; and 10 for application of ML methods to diseases,5 diseases are related, 2 articles for obesity, 4 articles for diabetes, 2 articles for cancer, 1 article for cardiovascular disease, and 1 article for others; and 4 references are about medical imaging using ML methods; in addition,1 reference study about gut microbiome and disease.

## 4. Discussion

Bibliometrics can help us find the hot topics, judge the research trend and reveal the key papers to guide the publication selection and scientific research.^[20]^ The current analysis of annual publication quantity and citation rate of obesity research using ML methods related articles from 2004 to 2023 demonstrates a significant rise in research intensity. Especially after 2020, the amount of publication and citation has increased significantly, with approximately 25 times as many publications in 2021 as in 2011, analyzing the reasons, it may come down to the breakthrough and development of AI, the development and integration of interdisciplinary disciplines, and the turnaround in the COVID-19 situation.

The increasing prevalence and youthfulness of obesity, which is strongly linked to many metabolic diseases and cardiovascular diseases, is a widespread concern.^[21]^ As ML and algorithms evolve, more and more scholars are conducting ML techniques for obesity research.^[6]^

### 4.1. Overview of publications

According to 3286 publications on obesity research utilizing ML methods from above 5000 institutions with over 20,000 authors in the vast WOSCC database during January 1, 2004 and December 31, 2023, this study conducted a bibliometric and visualization analysis.

In the country/region analysis, as displayed in Table 1 as well as Figure 3, The United States leads worldwide in both publication volume and frequency of citations, indicating that it has made a tremendous contribution to the development of this field, followed by China. Meanwhile, Australia has highest centrality of 0.17, followed by Canada, and USA ranks the third with a centrality of 0.15, demonstrating their core position in this research. The United States accounts for 5 out of the top 10 institutions with numerous publications, but the centrality is generally low, indicates that cooperation needs to be strengthened between each institution.

In terms of author, as seen in Table 3, Kim, Jong Yeol (Korea Inst Oriental Med, Korea) comes first in comparison to publications, Leo Breiman has the greatest co-citation rate, showing that he is a prominent figure in the field. Leo Breiman is a prominent American statistician and computer scientist, best known for his contributions to the fields of statistics and ML. He makes significant advancements in statistical methodology and is a key figure in the advancement of ML, including the development of the random forest algorithm, bagging, and contributions to decision tree.^[22]^

### 4.2. Hotspots and frontiers

Keyword analysis and highly cited reference analysis aid in clarifying current state and prevalent items of obesity research using ML methods. Analyzing the active research directions from the frequency of keyword appearances reveals that ML methods, obesity and its complications are in focus.

### 4.3. ML methods applied to obesity

ML are generally categorized into several methods and classification based on the learning paradigm, task, and nature of the data, such as supervised learning, semi-supervised learning, unsupervised learning, reinforcement learning, deep learning and so on. It has become a transformative force, ubiquitous and indispensable for resolving challenging issues in medical practice.^[23]^ ML can better assist doctors in diagnosing diseases and improve prognosis, especially in the area of image diagnosis involving radiology and pathology, in order to improve clinical services.^[24]^ In obesity research using ML methods, as the process of discovering patterns and knowledge from large amounts of data, “data mining” is continuous hot during 2007 to 2019, as well as “support vector machine,” as a supervised ML algorithm that is commonly used for classification and regression tasks, which is hot during 2009 to 2019, they are the frequently applied algorithm. As a type of deep learning algorithm commonly used in image recognition, “convolutional neural networks” is new, “deep learning” and “predictive models” have been frequently used during the study period, while deep learning is a subset of ML that deals with algorithms inspired by the structure and function of the human brain’s neural networks and predictive models, predictive models are ML models that are created to predict the outcome of a certain event or situation based on past data and patterns.

The highest cited reference is an article introducing R, a software environment and programming language intended for graphical representation, data analysis, and statistical computing.^[25]^ And of the top 25 references with the most explosive citations, the most highly cited is an article describing support vector machines.^[25]^ It shows that this algorithm is frequently utilized in the obesity research using ML methods. The fifth highly cited reference of the top 25 references with the most explosive citations, “Machine learning in Medicine,” Introduces some basic concepts in ML, explains the 2 forms of learning methods, namely supervised learning and unsupervised learning, then raises lists the problems and thoughts on ML, and illustrative examples of ML in medicine.^[1]^ Three papers are about deep learning of the top 25 references with the most explosive citations. Deep learning, a particular type of ML, employs artificial neural networks to mimic the intricate functionalities of the human brain. It involves training algorithms on large amounts of data including pictures, text, sounds, and others in order to learn and make predictions or decisions without explicit programming.^[26]^ In the future, deep learning remains a hotspot in obesity research using ML methods, it can enable more accurate diagnoses, personalized treatments, and faster drug discovery.

### 4.4. Obesity and its complications using ML methods

The keywords of diseases related to obesity research using ML methods mainly include “overweight,” “obesity,” “diabetes,”^[27]^ “metabolic syndrome,” “insulin resistance,” “cardiovascular disease,”^[28]^ “cancer,”^[29]^ “COVID-19,” et al, and “diabetic retinopathy” is hot after 2021, according to the Figure 8A and top 25 keywords with the most explosive citations. Obesity among children becomes widely concerned during 2011 to 2017, which may continue into adults, causing a variety of chronic diseases. The tenth highly cited reference of the top 25 references with the most explosive cited references, “Machine learning techniques for prediction of early childhood obesity,” explores the application of ML techniques to predict young children’s obesity, emphasizing the significance of early detection as well as discussing the implications for healthcare and preventive measures.^[30]^

In pathogenesis and therapeutic targets, gut microbiota, energy expenditure, genome wide association are research hotspots. Epidemiology, such as COVID-19 is hot after 2021, so obesity is associated with an epidemic. Diagnosis and treatment of obesity group with epidemic disease maybe the hotspot in the future.

In diagnosing obesity and its complications, ML techniques are extensively employed, predictive models in particular, MRI, and other imaging examinations.^[31,32]^ In terms of therapy, much research focuses on physical activity from 2011 to 2018. Hope more research focuses on drugs in this field in the future.

### 4.5. Limitations

Utilizing bibliometrics as a research tool and methodology, the research, for the very first time, reveals the current status, emerging trends, and areas of active research of obesity research employing ML methods over the last 20 years. However, this paper has some limitations for various reasons. Initially, a solitary database was examined in this study despite WoSCC being acknowledged as the primary publication source for bibliometric analysis. Second, the language of documents selected for this paper is limited; the fact that only English documents were chosen might have affected the results. Lastly, there can be bias in the study’s data; for instance, the names of the same institution may not be consistent across time.

## 5. Conclusion

With the development of technology and accumulation of data, ML has been increasingly used in the medical field, especially in disease diagnosis and prediction, personalized treatment, medical resource optimization, and new drug development. ML technology can identify obesity risk factors and early warning signals by analyzing a large amount of clinical data, helping doctors to detect patients’ Obesity problems early and develop personalized treatment plans. This study can help clinicians understand the hotspots and frontiers of obesity research, especially the application of ML as an emerging tool in obesity.

By bibliometric and visualization analysis of the publications in the last 2 decades, this study identified influential works and authors, assessed the quality and impact of journals, mapped the scientific landscape, contributed to a thorough comprehension of the significance and quality of studies, and informed academic research and scholarly communication about current general trends and frontiers on obesity research employing ML techniques. The study outcomes show that the key journals are *Scientific Reports*, *PLOS One*, *Nutrients*, etc. Leo Breiman is currently the most widely respected expert in the field. Deep learning, support vector machines, predictive models, gut microbiota, energy expenditure, and genome are currently prominent areas of interest in obesity research using ML methods. The connection between obesity and its complications, such as diabetic retinopathy, and the interaction between obesity and epidemiology, such as COVID-19, may become hot topics for upcoming research. Children, personalized food recommenders, and nutrigenetic models are also worthwhile topics for additional study.

## Acknowledgments

We thank all the people who offered help with this study. The authors have no conflicts of interest to disclose.

## Author contributions

**Conceptualization:** Xiao-wei Gong, Si-yu Bai.

**Data curation:** Xiao-wei Gong

**Formal analysis:** Si-yu Bai.

**Funding acquisition:** Jian-zhong Liu.

**Investigation:** En-ze Lei, Lian-mei Lin.

**Methodology:** Xiao-wei Gong, Si-yu Bai.

**Project administration:** Yao Chen, Jian-zhong Liu.

**Resources:** Yao Chen, Jian-zhong Liu.

**Software:** Xiao-wei Gong, En-ze Lei.

**Supervision:** En-ze Lei, Yao Chen, Jian-zhong Liu.

**Validation:** Xiao-wei Gong.

**Visualization:** Xiao-wei Gong.

**Writing – review & editing:** Xiao-wei Gong, Si-yu Bai, Yao Chen, Jian-zhong Liu.
